# CD8 T cell memory: it takes all kinds

**DOI:** 10.3389/fimmu.2012.00353

**Published:** 2012-11-27

**Authors:** Sara E. Hamilton, Stephen C. Jameson

**Affiliations:** Department of Laboratory Medicine and Pathology, Center for Immunology, University of Minnesota Medical SchoolMinneapolis, MN, USA

**Keywords:** protective immunity, CD8 T cells, immune memory, T cell differentiation, host-pathogen interactions

## Abstract

Understanding the mechanisms that regulate the differentiation and maintenance of CD8^+^ memory T cells is fundamental to the development of effective T cell-based vaccines. Memory cell differentiation is influenced by the cytokines that accompany T cell priming, the history of previous antigen encounters, and the tissue sites into which memory cells migrate. These cues combine to influence the developing CD8^+^ memory pool, and recent work has revealed the importance of multiple transcription factors, metabolic molecules, and surface receptors in revealing the type of memory cell that is generated. Paired with increasingly meticulous subsetting and sorting of memory populations, we now know the CD8^+^ memory pool to be phenotypically and functionally heterogeneous in nature. This includes both recirculating and tissue-resident memory populations, and cells with varying degrees of inherent longevity and protective function. These data point to the importance of tailored vaccine design. Here we discuss how the diversity of the memory CD8^+^ T cell pool challenges the notion that “one size fits all” for pathogen control, and how distinct memory subsets may be suited for distinct aspects of protective immunity.

## INTRODUCTION

Memory CD8^+^ T cells form a unique population, which are able to confer protection against many diverse pathogens. During acute infection, rare, naïve, antigen-specific T cell clones inhabiting secondary lymphoid tissue scan incoming pathogen-derived peptide–MHC I complexes. Once recognition of cognate peptide occurs, in conjunction with appropriate costimulatory and cytokine signals, CD8 T cells undergo massive proliferation and differentiation to form an effector cell population. Effector cells then utilize multiple mechanisms (predominantly cytolysis, IFN-γ, and TNF production) to destroy pathogen-infected cells. After the clearance of infection, a dramatic contraction phase ensues, leaving behind a small, extremely heterogeneous population of long-lived cells that compose the CD8 memory T cell pool (Harty and Badovinac, 2008; Jameson and Masopust, 2009). These cells remain at stable numbers, which are much higher than the starting, naïve population, in the absence of antigen or MHC class I, relying instead on survival cues from homeostatic cytokines IL-7 and IL-15 (Schluns and Lefrancois, 2003; Antia et al., 2005; Surh and Sprent, 2008). In addition to quantitative increases, memory T cells are qualitatively changed from their naïve counterparts, enabling them to respond to reinfection with faster, more robust activity. Recent evidence has revealed the extreme heterogeneity of the memory T cell pool contains, the cues that influence their formation, and the unique challenges which complex pathogens present. Here, we review recent advances, with special emphasis on identification of memory T cells capable of prompt control of acute pathogen infections, and the relevance for vaccine design.

## FACTORS INFLUENCING MEMORY T CELL FORMATION

CD8 memory T cell formation is influenced by multiple environmental cues that occur during priming. The combination of these factors regulates the size of the CD8 T cell response and the balance between memory and short-lived effector cell differentiation. Since this has been discussed in several reviews (Ahmed et al., 2009; Jameson and Masopust, 2009; Cui and Kaech, 2010; Rutishauser and Kaech, 2010), we will briefly review key factors that influence generation of CD8 memory cells, highlighting newer findings.

### INFLAMMATORY CUES

In addition to encounter with specific peptide/MHC molecules and costimulation (in the form of B7 or other ligands), the inflammatory environment surrounding the cell also has a large impact on the development of memory populations. IL-12 and IFN-α/β are well-defined for providing a “Signal 3,” and promote optimal development of both effector and memory cell populations (Mescher et al., 2006; Curtsinger and Mescher, 2010), although the specific cytokine may impact the characteristics of the resulting effector and memory pool. Signal 3 cytokines regulate an impressive number of gene expression changes (including those encoding factors that regulate survival, effector function, and trafficking) and chromatin remodeling may also be an important action of Signal 3 cytokines in CD8 T cells (Agarwal et al., 2009). Stimulatory signals generated during acute bacterial or viral infections can increase the number of effector cells generated during an immune response, but can also delay the onset of memory development (Badovinac et al., 2004, 2005). Cytokines TNF-α, IL-2, and IFN-γ have all been shown to impact the CD8 T cell response as naïve cells differentiate into memory, and limiting early inflammation favors the generation of memory cells (Badovinac et al., 2004, 2005; Harty and Badovinac, 2008). Overall, the data to date show that inflammation and the ensuing cytokine milieu can have a remarkably strong influence on the developing CD8 memory pool.

These effects of inflammation operate, at least in part, though changes in several key transcription factors. Multiple transcription factors (including T-bet, Eomesodermin, Blimp-1, Bcl-6, Id-2, Id-3, TCF, and Stat3) have been shown to modulate the development of short-lived effector versus memory cells, and the expression of several of these factors is influenced by the cytokine milieu (Joshi and Kaech, 2008; D’Cruz et al., 2009; Rutishauser and Kaech, 2010; Ji et al., 2011; Olson and Jameson, 2011; Yang et al., 2011; Xue and Zhao, 2012). These transcriptional regulators often operate in antagonist pairs (perhaps best defined for Blimp-1 and Bcl-6; Nutt et al., 2008; Crotty et al., 2010). A comprehensive picture of how transcription factors and epigenetic changes (Weng et al., 2012) coordinate with each other and additional signals to mold CD8 memory differentiation has yet to emerge – but manipulation of the cytokine environment offers a promising opportunity to regulate the balance between effector and memory CD8 T cell differentiation.

Recent data suggest an interesting twist in the impact of inflammatory cues on effector and memory differentiation. The chemokines CXCL9, CXCL10, and CXCL11 are effectively induced by IFN-γ, and strongly influence migration of CXCR3-expressing immune cells. Hence CXCR3 expression is important for T cell control of various pathogens. However, the ability of CXCR3 signals to retain activated T cells in sites of antigen presentation appears to promote their terminal differentiation toward short-lived effector cells: CXCR3 deficient CD8 T cells form greatly increased numbers of long-lived memory cells, with reduced contraction from the effector phase of the response (Hu et al., 2011; Kohlmeier et al., 2011; Kurachi et al., 2011). Hence, not only the direct response to inflammatory cytokines, but also the response to secondary cues (in this case CXCR3 ligands) may promote effector differentiation, potentially at the expense of memory cell generation.

### METABOLIC CONTROL

It is also possible to manipulate the number and type of memory cells formed through metabolic agents. Interestingly, mammalian target of rapamycin (mTOR), a metabolic kinase, has been shown to be a key regulator of CD8 T cells as well as other immune cells (Pearce et al., 2009; Araki et al., 2011). Treatment with rapamycin, an inhibitor of mTOR, has been known for some time to inhibit cellular proliferation and has been used in clinical settings. Recent evidence has shown that inhibiting mTOR will also increase the number of central memory T cells formed, enhancing trafficking to secondary lymphoid organs (Sinclair et al., 2008; Araki et al., 2011). Additionally, either treatment with rapamycin or knocking down components of mTOR during the early phase of the immune response, increases the quantity of memory cells that survive long-term, supporting the concept that mTOR is a component of CD8 memory T cell differentiation (Araki et al., 2009). Although the exact mechanism through which disruption of mTOR signaling enhances lymphoid CD8 T cell memory generation is currently unclear, the concept of capitalizing on differences between the metabolic states of effector versus memory (and naïve) T cells offers an interesting opportunity for therapeutic manipulation of CD8 T cell differentiation (Prlic and Bevan, 2009; van der Windt and Pearce, 2012).

### ANTIGEN RESTIMULATION

Repeated acute exposure to foreign antigen has a dramatic effect on the memory CD8^+^ T cell pool. Characteristics of the memory pool differ between the primary pool (generated by one round of antigen exposure) compared to “secondary” (or tertiary) memory cells induced by boosting. The differentiation of T_CM_ is considerably delayed, and cells bearing effector-like traits (including expression of KLRG1 and granzyme B) are maintained for considerably longer periods (Jabbari and Harty, 2006; Masopust et al., 2006). These differences alter the functional and trafficking characteristics of the memory pool – for example, the relative paucity of T_CM_ in the secondary memory pool limits their ability to traffic through lymph nodes (while the abundance of T_EM_ may enhance survey of peripheral tissues). Depending on the context of reinfection, such changes in localization could be either a benefit or detriment to the host – indeed Nolz and Harty (2011) propose that boosting may impair the ability of CD8 T cells to mount protective responses against certain pathogens (due at least in part to altered trafficking), while control of other pathogens is improved by boosting. Similarly, the number of antigen-specific cells is increased with boosting, and this can allow achievement of a threshold for protective immunity (perhaps most dramatically illustrated for the response to malaria; Schmidt et al., 2008) but this may come at the cost of the boosted memory cells’ capacity for proliferation after antigen re-exposure (Masopust et al., 2006; Wirth et al., 2010). Again, depending on the context of the response required, this trait may become a limitation for the immune response. Analysis of boosted memory T cells is important, partly for evaluating optimal vaccination strategies, and partly because pathogen reencounter is likely to occur in natural situations (unlike the artificially controlled exposure used in experimental studies or vaccination), and hence may be a better indication of normal immune function.

Furthermore persistent infections that periodically reactivate from a latent state (such as occur with several herpes viruses) can promote memory CD8 T cell “inflation,” producing T cells with the characteristics of boosted memory CD8 T cells (Snyder et al., 2008), and such features have been exploited for induction of protective immunity in models of HIV (Hansen et al., 2011). On the other hand, excessive or sustained antigen exposure (as occur during some chronic infections), can lead to the decline of CD8 T cell function – this has been reviewed extensively by others (Kaech et al., 2002; Virgin et al., 2009; Wherry, 2011), and hence will not be further explored here. However, this raises the important point that considerably more information is needed to understand the conditions that dictate whether multiple antigen encounters leads to enhancement versus impairment of the CD8 T cell response.

## HETEROGENEITY AMONG MEMORY CD8 T CELLS

The memory pool contains many distinct subsets of CD8 T cells with differing proliferative, survival, trafficking, and functional qualities (Seder et al., 2008; Jameson and Masopust, 2009). Elegant single cell transfer and bar-coding experiments show that an individual naïve TCR transgenic CD8 T cells is capable of forming diverse effector and memory populations (Stemberger et al., 2007; Gerlach et al., 2010), arguing against the model that distinct memory subsets are occupied by different clones, or cells receiving distinct initial activation cues.

Considerable work has gone into defining cell surface markers to subset the memory pool into functionally distinct populations, in both mice and humans (Seder and Ahmed, 2003; Seder et al., 2008; Masopust and Picker, 2012). Major classifications are discussed below. However, in contrast to the depth of information on the factors regulating effector versus memory differentiation, much less is known about the signals that drive appearance of distinct memory subsets.

### EFFECTOR AND CENTRAL MEMORY

The most widely characterized subset division is that of central and effector memory cells (Sallusto et al., 1999; Wherry et al., 2003), which are defined based on the coordinate expression of CCR7 and CD62L. Both molecules interact with components displayed on the high endothelial venules of lymph nodes – CD62L interacting with carbohydrate moieties termed lymph node addressins while CCR7 binds the “homeostatic” chemokines CCL19 and CCL21. Memory cells that express these two molecules are termed central memory (T_CM_), and efficiently traffic into lymph nodes, but are not predominant in peripheral tissues. In contrast, effector memory (T_EM_) cells do not express CCR7 or CD62L and are excluded from lymph nodes, but can be found in the spleen (especially in the red pulp; Jung et al., 2010) and are prevalent in non-lymphoid tissues (Masopust et al., 2001). In addition to these trafficking differences, the T_CM_ pool exhibits improved long-term survival and enhanced proliferation upon antigen restimulation, compared to the T_EM_ population, while the T_EM_ subset, especially cells isolated from tissues, show more rapid deployment of effector functions compared to T_CM_ (Kaech and Wherry, 2007; Jameson and Masopust, 2009).

The “T_EM_” subset is heterogeneous and can be further dissected. First, some CD62L^low^ CCR7^high^ cells have been defined (Unsoeld and Pircher, 2005): given that CCR7 supports T cell migration from some non-lymphoid tissues (Debes et al., 2005), this phenotype may be indicative of a specialized trafficking pattern. Furthermore, the general T_EM_ phenotype includes both a recirculating pool (with special predilection to migration through non-lymphoid tissues) as well as non-recirculating cells, termed resident memory cells, discussed next.

### RESIDENT MEMORY

More than a decade ago, seminal studies documented the existence of CD8 memory cells in diverse non-lymphoid tissues, in addition to their counterparts in lymphoid sites (Masopust et al., 2001; Masopust and Lefrancois, 2003). Memory CD8 T cells persist long-term in peripheral tissues, and were noted as having increased granzyme B expression and more potent killing capacity than central memory cells (Masopust et al., 2001; Marzo et al., 2007). Such cells were originally thought to be part of the recirculating T_EM_ pool (with which they share some key phenotypic traits), but more recent studies indicate that there is a distinct non-recirculating population of memory CD8 T cells, termed resident memory (T_RM_), in many tissues, including the IEL, skin, lung, brain, and salivary gland (Gebhardt et al., 2009; Masopust et al., 2010; Wakim et al., 2010; Jiang et al., 2012; Masopust and Picker, 2012).

T_RM_ cells have been identified at barrier surfaces in mice and non-human primates (Bevan, 2011; Sheridan and Lefrancois, 2011; Masopust and Picker, 2012), with similar cells characterized in human skin (Clark et al., 2012) and this pool is of interest as a critical first line of defense against infection. While there are numerous questions about the pathways involved in establishment and maintenance of T_RM_, the pool found in the mouse small intestine IEL pool is especially well-characterized. Although phenotypically related to T_EM_, the SI-IEL pool displays some distinct markers, including upregulated CD103 (the αE integrin chain, which, when paired with the β7 chain, is a receptor for E-cadherin) and CD69 (Sheridan and Lefrancois, 2011; Masopust and Picker, 2012). Surprisingly, despite the common association of CD69 with TCR stimulation, foreign antigen exposure is not required for generation of the SI-IEL pool, which can be induced by homeostatic mechanisms (Casey et al., 2012), arguing against an obligatory role for an antigen depot in sites occupied by T_RM_. Cytokines, including TGF-β, are important for induction of CD103 on SI-IEL T_RM_ cells, and CD103 itself is important for sustained residency of this population (Casey et al., 2012). It is not yet clear whether these requirements will apply to T_RM_ in all tissues, and whether additional cues are needed to initiate or sustain tissue residency, but these data highlight the sophisticated mechanisms which allow segregation of recirculating from tissue-resident cells.

### EFFECTOR-LIKE MEMORY CELLS

Another CD8 memory T cell division scheme was defined by Woodland and colleagues, based on CXCR3, CD27, and a glycosylated form of CD43 (Hikono et al., 2007). These markers further fragment the T_CM_ and T_EM_ pools, offering refinement of functional properties within the memory-stage pool, for example showing that CD27^hi^CD43^lo^ cells were superior over other subsets in their ability to proliferate after rechallenge (Hikono et al., 2007). CD27^lo^CD43^lo^ cells on the other hand, showed markers associated with the effector phase, including expression of KLRG-1 and granzyme B, and showed impaired proliferative responses. This “effector-like” population is maintained for many months following the response to respiratory infections (Hikono et al., 2007) as well as system infection with diverse pathogens (Olson et al., unpublished data). Such cells decline over time in the primary immune response: however, cells with this phenotype are maintained long-term and at high frequency following antigen-specific boosting (Olson et al., unpublished data) and, as will be discussed below, show optimal immediate protective control against acute bacterial and viral infections (Olson et al., unpublished data). Notably, this effector-like phenotype (characterized in lymphoid tissues) overlaps with the resident memory pool – for example, cells from the small intestinal IEL are CD27^low^, CD43^low^, granzyme B^high^ – although some other markers are distinct (e.g., SI-IEL cells are KLRG-1^low^ and CD69^+^ while effector-like cells in lymphoid tissues are KLRG-1^high^, CD69^-^). Hence the potential relationship between effector-like and T_RM_ cells needs to be investigated further.

### MEMORY STEM CELLS

Recent evidence has suggested that some memory T cells may have the ability to produce a specialized self-renewing population, sharing signaling pathways with hematopoietic stem cells. Gattinoni et al. (2009) proposed that induction of Wnt signaling suppresses Eomesodermin, and generates T cells of an unusual phenotype: CD44^low^, but high in expression of Sca-1, CD122, CD62L, and Bcl-2, which bears features of proposed “Memory Stem Cells” (first identified in a transplant setting; Zhang et al., 2005). This change allowed T cells to go through many more cell divisions than normal, as well as proliferate and differentiate in response to antigen. This property was shown to be beneficial in a tumor model, suggesting the possibility that antigen-specific memory stem cells (even in small numbers) may be a useful immunotherapy tool (Gattinoni et al., 2009; Koehn and Schoenberger, 2009). However, other studies, using similar approaches, concluded that activation of the Wnt signaling pathway in mouse CD8 T cells did not promote production of a memory stem cell pool, but rather may attenuate initial naïve response (Driessens et al., 2010; Prlic and Bevan, 2011). Hence, considerable controversy currently surrounds the definition of this memory subset. Nevertheless, the concept of a specialized memory stem cell, paired with the finding that humans possess a similar CD8 memory T cell subset with the ability to survive and reconstitute the T cell pool after depletion events like chemotherapy (Turtle et al., 2009; Gattinoni et al., 2011), will certainly provoke continued research and interest.

## WHICH SUBSET(S) OF MEMORY CD8 T CELLS OFFER OPTIMAL PROTECTION AGAINST PATHOGENS?

**FIGURE 1:**
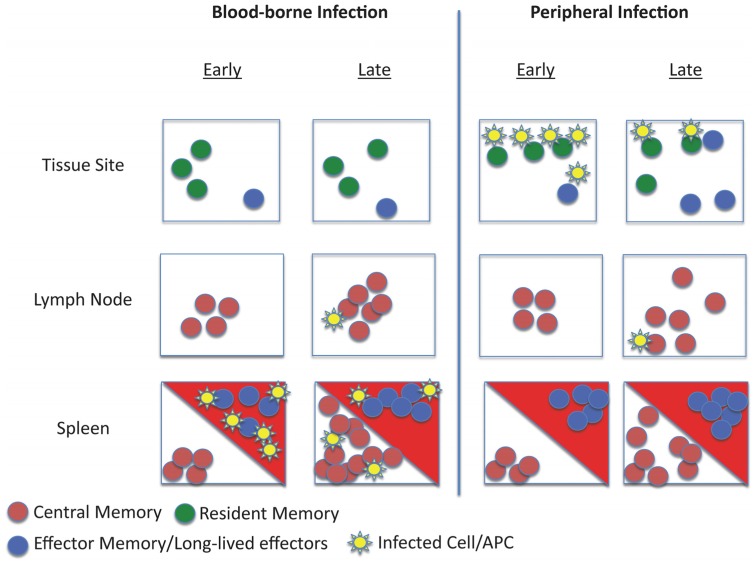
**Schematic of memory T cell distribution and reactivation during systemic and local infection**. The model depicts the response to infection of animals with pre-existing memory CD8 T cell populations. Different populations of memory cells occupy distinct niches: T_CM_ are restricted to lymphoid tissues, recirculating T_EM_ and long-lived effector-like cells traffic through tissues and certain lymphoid sites (such as the splenic red pulp – red triangles) while T_RM_ are strictly limited to tissue parenchyma. Upon infection via the blood (such as bacterial sepsis), pathogens may initially be controlled by effector-like and T_EM_ populations at entry sites (e.g., the splenic red pulp and marginal zone). Subsequently, activation and clonal expansion of T_CM_ increases the frequency of secondary effector cells. In this scenario, T_RM_ are not engaged in the response. However, during a peripheral infection (for example, viral infection in the skin), initial pathogen control is mediated by the T_RM_ pool. This inflammatory response may recruit circulating T_EM_ (and, potentially T_CM_) to the site of infection. Subsequent responses initiated in draining lymphoid tissues would control systemic spread.

A fundamental feature of the adaptive immune system (and the primary goal of vaccines) is that immune memory results in improved protection against pathogen reinfection. While this can, in part, be ascribed to the numerical increase in antigen-specific T cells that follows immunization, heterogeneity within the memory T cell pool naturally leads to the question of whether some populations of memory cells are better than others at protective immunity against a given pathogen (**Figure 1**).

Some years ago, the answer seemed relatively clear: T_CM_ had numerous features suggesting these were the critical memory population for long-term protective immunity. First, T_CM_ show very effective long-term maintenance, becoming the predominant memory subset over time following a primary antigen encounter. In addition, T_CM_ exhibit optimal recall proliferative capacity and the ability to quickly differentiate into potent effector cells upon antigen re-encounter. Finally, direct comparisons between T_EM_ and T_CM_ following infection with various pathogens (acute and chronic LCMV, vaccinia virus) suggested the T_CM_ pool was, overall, the superior subset for pathogen elimination (Wherry et al., 2003; Laouar et al., 2008). As discussed earlier, the representation of T_CM_ changes with antigen-specific boosting, which decreases the frequency of T_CM_ and delays their appearance: indeed, with heterologous prime/boost strategies, the frequency of T_CM_ can become quite low, with the antigen-specific memory CD8 T cell population dominated by T_EM_ and effector-like cells (Jabbari and Harty, 2006; Masopust et al., 2006; Wirth et al., 2010). This might lead to the conclusion that boosting the immune response, though clearly of benefit for high affinity B cell responses, could degrade the protective capacity of the CD8 memory pool. In fact, recent studies argued that boosted (or secondary memory cells) are indeed compromised for control of chronic LCMV and MHV infection, although responses to some other pathogens (*Listeria*, vaccinia, and acute LCMV infection) were unchanged or improved by boosting (Nolz and Harty, 2011).

On the other hand, additional studies suggested that cells of T_EM_ phenotype exhibited optimal pathogen control against some of the same pathogens (e.g., vaccinia virus; Bachmann et al., 2005) and against other systemic infections (e.g., *Listeria*; Huster et al., 2006). Furthermore, T_EM_ phenotype cells induced by heterologous prime/boosting were associated with improved protection against mucosal SIV challenge, with important implications for vaccination against HIV infection (Hansen et al., 2009, 2011).

Furthermore, in recent studies we examined the protective capacity of “effector-like” CD8 T cells that persist into the memory phase during primary responses and are the predominant antigen-specific pool following certain prime-boost strategies (Olson et al., unpublished data). These cells, bearing the phenotype of CD27^low^, CD43^low^, KLRG-1^high^, and CD127^int^ were poor at recall proliferation compared to subsets containing the classic T_EM_ and T_CM_ populations (Hikono et al., 2007; Olson et al., unpublished data): yet these effector-like cells mediated optimal protective immunity against *Listeria* and vaccinia infection at least in part due to preferential utilization of cytotoxic mechanisms (Olson et al., unpublished data). Since this effector-like subset shares some phenotypic traits with typical T_EM_ cells, care must be taken in evaluating data on the protective capacity of the T_EM_ subset. It is interesting to note that the rapid prime-boost strategy described by Harty and colleagues, which leads to highly efficient protection against various viral, bacterial, and parasitic infections (Pham et al., 2010), predominantly induces a CD27^lo^, CD43^lo^, KLRG1^hi^, CD127^int^, effector-like population, which persist long-term (Olson et al., unpublished data). Hence, this population – which might also be termed “long-lived effectors” to contrast with their short-lived counterparts found in the early immune response – represents an appealing goal for vaccination against certain diseases.

However, a limitation on many studies testing the protective capacity of distinct memory subsets is that they typically involves isolation of cells from lymphoid tissues followed by adoptive transfer into the blood. This approach neglects the T_RM_ populations existing in non-lymphoid sites, which (by definition) are not part of the recirculating pool found in lymphoid tissues. Experimentally, this issue is compounded by the finding that T_RM_ are inefficient at homing back to non-lymphoid tissues in the absence of restimulation (Masopust et al., 2010; Masopust and Picker, 2012). However elegant approaches, including parabiosis and selective depletion strategies have been used to test the capacity of T_RM_ to mediate protective immunity in non-lymphoid tissues. For example, Jiang et al. (2012) examined a parabiotic mice mouse model in the context of vaccinia infection in the skin: mice that contained both antigen-specific T_RM_ and recirculating memory cells rapidly cleared the infection, while mice with recirculating memory CD8 T cells alone showed impaired clearance of the virus. Other studies limited the capacity of recirculating memory cells to contribute to pathogen control, and again saw efficient protection mediated through T_RM_ (Hofmann and Pircher, 2011; Mackay et al., 2012). These data highlighted that in the context of a pathogen invasion at an epithelial surface, resident memory cells are superior to central memory or naïve CD8 T cells. Likewise, it is likely (although not proven) that the mucosal T_EM_-like CD8 T cells that offer optimal control of SIV infection (Hansen et al., 2011) are in fact T_RM_ (Masopust and Picker, 2012). Such data suggest that the T_RM_ pool is critical for first-line defense against infection at barrier surfaces, but presumably play a more minor role in responses to blood-borne infections (**Figure 1**).

## CONCLUDING REMARKS

The goal of vaccination is to rapidly control infection to prevent or minimize the occurrence of disease. Determining the CD8 memory T cell(s) best able to achieve that goal is critical for future development of effective vaccines as we move to apply bench work to the clinic. Defining a “protective” memory cell is always context dependent. Is the infection acute or chronic? What is the inflammatory environment created? What is the life style of the pathogen and its location in the host? These factors and others impact the developing CD8 T cell response and should be at the forefront of our attempt to create the most useful memory T cell pool by vaccination. Thus, while it is tempting to try to define “The” optimal subset of memory CD8 T cells for protective immunity, the very fact of memory heterogeneity suggests that this diversity is useful for the immune system in different contexts: so, while rapid recall proliferation of a small T_CM_ memory subset may be suitable for control of chronic LCMV infection (Wherry et al., 2003; Nolz and Harty, 2011), very high numbers of T_EM_ and effector-like cells may be important for rapid control of liver-stage malaria infection (Schmidt et al., 2008; Pham et al., 2010) and establishment of a mucosal pool of T_RM_ may be essential for control of SIV (Hansen et al., 2011; Masopust and Picker, 2012). This discussion also raises the question of how quantity versus quality of antigen-specific memory CD8 T cells relates to protective immunity: while ideal immunity may produce a high frequency of diverse memory subsets, practical limitations force consideration of how many memory cells, of what type and in what locations, are sufficient for protection against a given pathogen. Careful analysis of the protective function mediated by various memory CD8 T cell subsets in distinct locations may provide suggest more streamlined vaccine approaches. Lastly, this review focuses on responses to infectious disease, but there may be quite different criteria for protective immune responses against tumors – indeed there is suggestive evidence that the self-renewing “memory stem cell” pool has key features for sustained responses against the self-antigens often targeted for cancer immunotherapy (Gattinoni et al., 2009; Koehn and Schoenberger, 2009).

Thus we propose two central challenges for optimizing protective CD8 T cell vaccination – first, defining the traits (including function and localization) which characterize protective CD8 T cell subsets (i.e., the correlates of protection); second, developing refined vaccination techniques to optimize production of the relevant subsets. As usual, the natural characteristics of the immune response (for example, the changes in memory populations induced by primary versus boosted immune responses) form a useful guide as to what can be achieved – but the pressing need is for vaccines against pathogens (such as HIV, malaria, tuberculosis) that elicit inefficient protective responses, hence radical strategies may be needed to achieve radical results.

## Conflict of Interest Statement

The authors declare that the research was conducted in the absence of any commercial or financial relationships that could be construed as a potential conflict of interest.
